# Deregulation of Bone Forming Cells in Bone Diseases and Anabolic Effects of Strontium-Containing Agents and Biomaterials

**DOI:** 10.1155/2014/814057

**Published:** 2014-03-31

**Authors:** Shuang Tan, Binbin Zhang, Xiaomei Zhu, Ping Ao, Huajie Guo, Weihong Yi, Guang-Qian Zhou

**Affiliations:** ^1^Key State Laboratory for Biotherapy, Sichuan University, Chengdu 610065, China; ^2^Anti-Ageing and Regenerative Medicine Centre, Shenzhen University, 3688 Nanhai Avenue, Shenzhen, Guangdong 518060, China; ^3^Institute for Systems Biomedicine, Shanghai Jiaotong University, Shanghai 200240, China; ^4^Department of Spinal Surgery, Nanshan People's Hospital, Shenzhen 518000, China

## Abstract

Age-related bone loss and osteoporosis are associated with bone remodeling changes that are featured with decreased trabecular and periosteal bone formation relative to bone resorption. Current anticatabolic therapies focusing on the inhibition of bone resorption may not be sufficient in the prevention or reversal of age-related bone deterioration and there is a big need in promoting osteoblastogenesis and bone formation. Enhanced understanding of the network formed by key signaling pathways and molecules regulating bone forming cells in health and diseases has therefore become highly significant. The successful development of agonist/antagonist of the PTH and Wnt signaling pathways are profits of the understanding of these key pathways. As the core component of an approved antiosteoporosis agent, strontium takes its effect on osteoblasts at multilevel through multiple pathways, representing a good example in revealing and exploring anabolic mechanisms. The recognition of strontium effects on bone has led to its expected application in a variety of biomaterial scaffolds used in tissue engineering strategies aiming at bone repairing and regeneration. While summarizing the recent progress in these respects, this review also proposes the new approaches such as systems biology in order to reveal new insights in the pathology of osteoporosis as well as possible discovery of new therapies.

## 1. Introduction 

Bone remodeling is a physiological process that maintains the integrity of the skeleton by removing old bone and replacing it with young matrix. An imbalance between bone resorption and bone formation with ageing will result in the increased rate of bone turnover rate and bone loss. The age-related progressive bone loss is exaggerated in patients with osteoporosis, a disease characterized by decreased bone mass, increased bone fragility, and increased risk of fractures [1]. As the elder population in the society rapidly increases, osteoporosis has become one of the most common public health problems.

In the case of the age-related bone loss or osteoporosis, the osteoblast-mediated bone formation is severely impaired [1, 2] due to decreased number and activity of individual osteoblastic cells. Such dysfunctions of osteoblasts may be caused by extrinsic mechanisms, such as changes in levels of systemic hormones and growth factors of bone tissues, and intrinsic mechanisms such as cellular apoptosis and senescence [2–4]. As a consequence, both trabecular and periosteal bone formation decline [5]. Most of the currently available therapies for osteoporosis, including amino-bisphosphonates, estrogens and selective estrogen receptor modulators (SERMS), and inhibitors for the receptor activator of nuclear factor *κ*B ligand (RANKL), take effect mainly by reducing bone resorption. However, these therapies frequently exhibit secondary effects due to the coupling phenomenon of bone formation by osteoblasts and bone resorption by osteoclasts [6]. Therefore, anabolic drugs are hoped to target osteoblastic cells to increase bone formation and bone strength [7], though anticatabolics may be efficient in stabilizing bone mass.

In this paper, accordingly, we focus on the functional regulation of osteoblast lineage cells in health and osteoporosis, the currently proposed anabolic agents such as teriparatide (PTH(1–34)), sclerostin or DKK1 inhibitors, and strontium that target specific signaling mechanisms underlying the osteoblast function and osteoporosis. The wide use of strontium in various orthopaedic scaffolds is also summarized. Through the analysis of current knowledge, some insights into further studies of osteoblast regulation and therapeutic exploration are provided.

## 2. Osteoprogenitors, Osteoblasts, and Osteocytes

Bone formation is dependent on the recruitment of sufficient number of osteoblasts and activity of individual osteoblasts. Osteoblastic cells are recruited to bone forming surfaces mainly from a group of skeletal stem cells with osteogenic differentiation potential. Bone marrow contains a small population of mesenchymal stem cells (MSCs) that are capable of giving rise to bone, cartilage, fat, or fibrous connective tissue [8]. Cell populations with properties characteristic of bone marrow MSC have been isolated from many other tissues such as adult peripheral blood, dental pulp, adipose tissue, fetal cord blood, and liver. These self-renewing multipotent stem cells can give rise to osteoprogenitor cells in various tissues under the right environmental conditions. Osteoprogenitor cells in turn give rise to and maintain the osteoblasts that synthesize new bone matrix on bone forming surfaces, the osteocytes within bone matrix that support bone structure, and the protective lining cells that cover the surface of quiescent bone. Therefore, osteoblastic lineage cells comprise a diverse population of cells, including immature, differentiating, and mature matrix-producing osteoblasts. Different osteoblasts may express different sets of genes, which may represent the heterogeneity of trabecular microarchitecture at different anatomic sites, the site-specific differences in the response to different signals and disease states, and regional variation in the ability to response to antiosteoporotic agents [9].

Particularly, flattened bone-lining cells are thought to be quiescent osteoblasts that form the endosteum on trabecular and endosteal surfaces and underlie the periosteum on the mineralized surface. More primitive osteoblasts that are found near functioning osteoblasts in the bone remodeling unit can be identified based on their expression of alkaline phosphatase. Active and mature osteoblasts synthesizing bone matrix have large nuclei, enlarged Golgi structures, and extensive endoplasmic reticulum. These osteoblasts secrete type I collagen and other matrix proteins vectorially toward the bone formation surface. Besides, osteoblasts have several other important roles in bone remodeling, including production of osteoclastogenic factors and bone mineralization [10]. Preosteoblasts encompass all cells transitioning from progenitors to mature osteoblasts and therefore are, by definition, hetero geneous. However, they are usually considered to express the transcription factor Runx2, at a more advanced stage of differentiation, both Runx2 and osterix (OSX), which both control the osteodifferentiation [11]. The differentiation stage of osteoblasts influences their functional roles in bone remodeling. Mice deficient in osteoblasts are deficient in osteoclasts [11]. In contrast, conditional depletion of mature osteoblasts* in vivo* only ablates bone formation and osteoclastic bone resorption persists [12]. Therefore, immature osteoblasts also influence osteoclastogenesis whereas mature osteoblasts perform the matrix production and mineralization functions.

During bone formation, a subset of osteoblasts undergoes terminal differentiation and becomes engulfed by unmineralized osteoid [13]. Following mineralization of the bone matrix, these entombed cells are called osteocytes. Osteocytes are cocooned in fluid-filled cavities (lacunae) within the mineralized bone and are highly abundant, accounting for 90–95% of all bone cells [13]. Osteocytes have long dendrite-like processes extending throughout canaliculi (tunnels) within the mineralized matrix. These dendrite-like processes form a network and interact with other osteocytes and with osteoblasts on the bone surface [14]. The primary function of the interaction between the osteocyte-osteoblast/lining cell syncytium is mechanosensation [15]. Osteocytes transduce stress signals from bending or stretching of bone into biologic activity and respond to mechanical load. The network is thought to be integral in the detection of mechanical strain and associated bone microscopic cracks/fractures within the mineralized bone that accumulates as a result of normal skeletal loading and fatigue [16]. Signaling molecules involved in mechanotransduction include prostaglandin E2, cyclooxygenase 2, various kinases, Runx2, and nitrous oxide. Therefore, osteocytes initiate and direct the subsequent remodeling process and support bone structure and metabolism.

Osteocytes express osteocalcin, galectin 3, CD44, and several other bone matrix proteins that support intercellular adhesion and regulate exchange of mineral in the bone fluid within lacunae and the canalicular network. Osteocytes regulate phosphate metabolism and matrix mineralization through the secretion of phosphate-regulating factors such as FGF23, Phex, Dmp1, and expression of sclerostin (encoded by gene SOST) and DKK1 that negatively regulates Wnt and BMPs signaling [17]. Osteocytes are linked metabolically and electrically through gap junctions composed primarily of connexin 43, which are required for osteocyte maturation, function, and survival [18].

## 3. The Molecular Regulation of Osteoblast Differentiation and Function

Differentiation of mesenchymal stem cells into the osteoblast lineage is under tight regulation orchestrated through multiple signaling pathways. Among the well-characterized are the fibroblast growth factor (FGF), transforming growth factor *β* (TGF*β*), hedgehog families, Wnt signaling, and notch pathways [19].

### 3.1. The FGF Signaling

The FGF pathway consists of 23 ligands that transduce their signal through one of the four FGF receptors (FGFRs), functioning in initiating condensation of the mesenchyme and proliferation of progenitor cells. Upon binding to cell surface receptors, FGFs lead to activation of multiple signaling modules, including mitogen-activated protein kinase (MAPK), phosphoinositide 3-kinase (PI3K), STAT1, and PKC. Temporal expression and activity of FGFR are critical for membranous bone formation and regulate the proliferation, differentiation, and apoptosis of osteoblasts [20]. FGFRs contribute to normal skeletal development, as gene mutation or deletions are associated with severe dwarfism [21]. FGF18 plays a critical role in maturation of osteoblast and FGF2 increases RUNX2 phosphorylation and functional activity [22]. Similarly, the activation of FGFR2 signaling results in increased Runx2 expression and enhanced osteoblast differentiation [22]. FGFR1 signaling at an early developmental stage promotes osteoblast differentiation without affecting Runx2 expression. Finally, FGFR3 is involved in regulation of osteoblast number and osteoid mineralization. Therefore, FGF signaling has diverse roles in the regulation of preosteoblast proliferation and osteoblast differentiation through different receptors.

### 3.2. BMPs

Bone morphogenetic proteins (BMP) are members of the TGF*β* superfamily. This group of proteins has a number of diverse functions in multiple developmental processes ranged from embryogenesis, organogenesis, bone formation, cell proliferation, and stem cell differentiation [23–28]. BMPs signal through homomeric or heteromeric type I and type II receptors, which are expressed in all cell types. Specific BMP receptors influence specific lineage direction. BMP2 signaling is required for the stimulation of mesenchymal progenitor cells by inducing expression of both Runx2 and Osterix, leading to osteoblast differentiation [29–31]. Induction of Runx2 and Osterix by BMP2 and subsequent upregulation of osteoblast-specific genes involves Dlx5, Smad transducers, and the MAPK pathway. TGF*β* itself plays more complex role during bone remodeling, with the inhibition of Runx2 and osteoblast differentiation* in vitro* but mainly promoting bone formation* in vivo* [29, 32].

### 3.3. The Wnt Signaling

During skeletal development, the Wnt signaling is implicated in multiple steps and processes, including proximal-distal outgrowth and limb patterning, and in MSC lineage commitment for chondrogenesis, osteogenesis, myogenesis, and adipogenesis [33–36]. Consequently, the Wnt signaling affects all aspects of skeletal development. The importance of Wnt signaling in bone diseases has been recognized since the rare human mutations affecting bone negatively (osteoporosis-pseudoglioma syndrome) or positively (high-bone mass phenotype) were all identified to reside in components of the canonical Wnt signaling machinery a decade ago. Mouse genetic studies have further confirmed the role of the pathway in the regulation of bone homeostasis, with activation of the pathway leading to increased, and inhibition to decreased, bone mass and strength [37] and the Wnt signaling is now known as a key mechanism regulating bone metabolism [38]. Activation of the Wnt signaling results in a generalized increase in bone mass throughout the skeleton. Wnt ligands activate numerous intracellular pathways upon targeting on various membrane receptors, which are either dependent or independent on *β*-catenin, an intracellular transducer. *β*-catenin is expressed in mesenchymal precursor cells and its inactivation promotes their differentiation into chondrocytes instead of osteoblast [34]. During the *β*-catenin-dependent Wnt signaling, *β*-catenin is stabilized following binding to its receptors Frizzled (FZD) and lipoprotein receptor-related protein 5 (LRP5) or LRP6, which in turn lead to the transcription of target genes such as Runx2. On the basis of extensive studies conducted thus far on Wnt signaling in bone, the pathway has now become the target for therapeutic intervention to restore bone strength in millions of patients at risk for fracture [37].

### 3.4. Hedgehog

The hedgehog signaling through Indian hedgehog (Ihh), a secreted molecule of the hedgehog family, is required for osteoblast differentiation through endochondral bone formation[39]. Ihh binds to the receptor patched homologue 1 (PTCH1) through the transmembrane protein smoothened (SMO), consequently regulating target gene transcription. Ihh controls osteoblast differentiation firstly by inducing Runx2 in mesenchymal cells. Secondly, Ihh enhances Runx2 action through an interaction between signal transducer Gli2 and Runx2 in osteoblast [40]. Ihh is also needed for osteoblast proliferation and survival and mice deficient Ihh gene lack osteoblast progenitor cells [39].

### 3.5. The Notch Signaling

The notch signaling mediates broad cell-cell communications. Once their ligands are binding to the neighbouring cell surface, notch receptors are cleaved by the *γ*-secretase complex. Consequently, the intracellular domain of notch is released from the plasma membrane and translocates to the nucleus, interacting with RBPJ*κ*/CBF1 to activate downstream target transcription factors. By physically associating with Runx2 and interfering with functional activity of Runx2, notch inhibits osteoblast differentiation. Mutations in the notch signaling cause skeletal patterning defects in human and notch deficiency leads to severe osteoporosis in mice [41]. Interestingly, through its expression in osteoblasts, notch exerts dimorphic effect during bone remodeling; notch also inhibits osteoclast differentiation through controlling production of “decoy” receptor OPG by osteoblast [41]. Thus far, the role of the notch signaling in bone diseases remains to be further elucidated.

### 3.6. Hormones

Besides local growth factors, a number of systemic hormones regulate bone mass by regulating osteoblast differentiation and influencing bone formation. Many of them act on osteoblasts to express M-CSF and RANKL that in turn regulate osteoclast differentiation. We herein only discuss some as examples, particularly those understood well thus far.

Parathyroid hormone (PTH) produced by the chief cells of parathyroid gland plays a primary role in calcium homeostasis through its action on bone and kidney and through enhanced synthesis of another hypercalcemic hormone, 1,25(OH)2 vitamin D3 [42]. The anabolic effects of PTH on bone formation are mediated through PTH receptor-dependent mechanisms. PTH enhances osteoblastic cell proliferation and function, extends mature osteoblast life span through antiapoptotic effects, enhances the Wnt signaling through inhibition of Wnt antagonist sclerostin, and promotes the local production of bone anabolic growth factors such as insulin-like growth factor 1 (IGF1) [43]. In addition, PTH promotes bone formation partially through phosphorylation and activation of Runx2, resulting in expression of osteoblast genes [44]. PTH also inhibits proteasome mediated degradation of Runx2 and increases expression of osterix to enhance osteoprogenitor lineage determination [45]. As the only approved anabolic agent, intermittent PTH therapy has been demonstrated to have beneficial effects on increasing bone mass and diminishing bone fragility associated with osteoporosis resulting from aging, sex hormone deficiency, and glucocorticoids use [46]. The other major hypercalcemic hormone is 1,25(OH)2 vitamin D3, a steroid hormone that favors intestinal absorption of calcium [47]. Deletion or inactivation of the vitamin D receptor (VDR) in mice and in humans leads to rickets, a phenotype completely reversible in both organisms by treatment with calcium. Vitamin D3 positively regulates the expression of osteoblastic phenotype markers. However, continuous exposure to parathyroid hormone (PTH), parathyroid hormone-related protein (PTHrP), and low doses of 1,25-dihydroxyvitamin D3 also stimulates osteoblasts to express M-CSF and RANKL, leading to increased osteoclast production and bone resorption [48, 49].

An important nonsteroidal regulator of bone mass is leptin, a hormone that functions through an inhibitory action of the hypothalamus on bone formation [50, 51]. Leptin is made by fat cells and functions to suppress appetite and inhibit bone formation by binding to receptors in the hypothalamus. Mice and humans deficient in leptin or its hypothalamic receptor are obese and have a higher than normal bone mass. The effector pathway from hypothalamus to bone is the sympathetic nervous system and sympathetic neurons produce noradrenaline, which binds to *β*2-adrenergic receptors (*β*2-AR) on osteoblasts [51, 52]. Mutant mice lacking *β*2-AR have increased bone mass but do not respond to leptin by reduction in bone mass [53], while ovariectomized *β*2-AR null mice fail to lose bone mass, suggesting that maintenance of the sympathetic nervous system in bone may require estrogen, which is essential in regulating bone remodeling via two related receptors, ER*α* and ER*β* expressed by osteoblasts.

## 4. Deregulation of Bone Forming Cells in Diseases

The correct balance between bone deposition and resorption is crucial for the proper maintenance of the bone mass and the loss of this coupling is the starting point for osteoporosis [54]. This is a systemic, skeletal disorder characterized by low bone mass with a high susceptibility to fractures. Osteoporosis is characterized by reduced bone mass and deterioration of bone microarchitecture, resulting in bone fragility. Primary osteoporosis is either a postmenopausal or age-related disease of elderly people, essentially occuring because the production of bone by means of osteoblasts cannot compensate for bone resorption by osteoclasts [55]. Sex hormones, including both estrogen and androgen, act on osteoblasts for their survival and, at the same time, induce osteoclast apoptosis through activation of the FASL/FAS pathway [56]. Therefore, withdrawal or decline of sex hormones is the principal determinant of primary osteoporosis. Moreover, estrogens suppress the production of proosteoclastogenic cytokines (such as IL-1, IL-6, TNF-*α*, and RANKL) and stimulate the secretion of OPG by osteoblasts [57, 58].

### 4.1. The Activation of the Inflammatory Pathways

Many chronic diseases have a local or a systemic inflammatory basis, which has overall deleterious effects on bone mass [59, 60], leading to the secondary osteoporosis with an onset at any age. The stimulatory action of the NF-*κ*B signal transduction in osteoclast development and functional activity is widely recognized [61]; recently it has also been demonstrated that NF-*κ*B activation is potently inhibitory to osteoblast commitment, differentiation, and mineralization* in vivo* and* in vitro* [62]. Tumor necrosis factor (TNF-*α*) is a potent NF-*κ*B inducer and activation of p65 (a NF-*κ*B subunit) by TNF-*α* has been shown to suppress transcription of osteocalcin in osteoblastic cells [63]. Pharmacological suppression of TNF-*α* is reported to reverse age-related defects in bone formation in a mouse fracture healing model and synergizes TGF*β*- and BMP-2-induced Smad signaling in differentiating osteoblasts [64]. In addition, TNF-*α* upregulates Smurf1, an E3 ligase that promotes proteasomal degradation of bone morphogenetic signaling proteins [65]. NF-*κ*B signaling in osteoblasts has been shown to upregulate Smad7, a general inhibitor of Smad pathway. Finally, a direct inhibitory action of NF-*κ*B on bone formation was demonstrated* in vivo* to show time- and stage-specific inhibition of IB kinase (IKK) in differentiated osteoblasts, increased trabecular bone mass, and ameliorated ovariectomy-induced bone loss [66].

### 4.2. Other Signaling Alterations and Therapeutics Opportunities

Alterations in the Wnt signaling have profound impact on age-related bone loss in mice [67]. Mechanical loading upregulates the Wnt signaling in MSC [68], suggesting that the combination of reduced *β*-catenin signaling and decreased mechanical stimulation with age may contribute to the age-related decline in bone formation. Based on these and other studies, the important role of the Wnt signaling in the control of bone formation has been well recognized as this pathway is suggested to be a potential therapeutic target [69]. The Wnt signaling alteration is related to increased marrow adipogenesis. With the aim of increasing osteoblastogenesis and the bone formation, several pharmacological agents have been developed that act on bone marrow MSC to favor osteoblastogenesis and decrease adipogenesis [70]. Nonpharmacological means to enhance MSC differentiation toward osteoblasts include low-magnitude mechanical signals [71].

Most drugs currently available for the treatment or prevention of osteoporosis are antiresorptive, including estrogens and selective estrogen receptor modulators, bisphosphonates, and denosumab blocking the RANKL/RANK pathway [72]. It is desirable to identify novel agents that can exhibit an anabolic function in order to improve and restore bone mass. Teriparatide (PTH1-34) is currently the only US Food and Drug Administration- (FDA-) approved anabolic agent for the treatment of osteoporosis [37]. Although the effect of PTH can be anabolic or catabolic depending on the dose, intermittent administration increases trabecular bone formation [73, 74]. Systemic administration of antagonists to DKK1 or sclerostin may possibly affect only the skeleton, favoring the endogenous Wnt signaling and increasing bone formation without affecting the Wnt signaling in other organs.

Currently, only strontium ranelate proves to have roles in both the osteoclast inhibition and bone formation promotion. Therefore, further research in the topic may provide insights not only into improving the effects of Sr-containing agents but also into discovering novel and more effective therapies.

## 5. Anabolic Effects of Strontium-Containing Agents on Osteoblasts

Several strontium-containing agents, such as Sr fructose, 1,6-diphosphate, and strontium citrate [75], have been experimentally demonstrated to have antiosteoporotic effects, among which strontium ranelate is an approved drug [76–78].

At the cellular level, it was shown that bone marrow MSC culture, when exposed to strontium (Sr), displayed a significant increase in the expression of the master gene and Runx2, as well as bone sialoprotein (BSP), and this was associated with a significant increase in the formation of colony-forming unit osteoblasts (CFU-obs). Interestingly, the activation of gene expression by Sr varies with the differentiation stage of MSC: Runx2 and BSP in bone marrow MSC; Runx2 and osteocalcin in preosteoblasts; BSP and osteocalcin in mature osteoblasts. Strontium ranelate-treated ovariectomised (OVX) animals exhibited increased bone formation and decreased bone resorption, leading to prevention of trabecular bone loss and improvement of bone microarchitecture and strength [79–81]. Clinical data also revealed that strontium ranelate treatment increased bone mineral apposition rate and improved trabecular microarchitecture in postmenopausal osteoporotic women [82], which was associated further with reduced fracture risk [76, 78].

*In vitro* experiments showed that Sr ranelate had positive effects on osteoblastogenesis and activity of primary rat and human osteoblasts [83]. On the one hand, strontium enhanced the replication of preosteoblastic cells [84, 85] and reduced osteoblast apoptosis [86, 87]. On the other hand, strontium was found to activate many osteoblast differentiation markers, such as alkaline phosphatase, type-1 collagen, bone sialoprotein and osteocalcin in murine bone marrow MSC, osteoprogenitor cells, and immature osteoblasts [80, 87, 88]. Furthermore, the agent also promoted the ultimate differentiation of human osteoblasts into osteocytes, as indicated by the increased expression of osteocyte-restrictive markers such as dentin matrix protein 1 [85]. Overall, the available* in vitro* data indicate that strontium promotes the osteogenic differentiation program and reduces osteoblast apoptosis, thereby promoting osteoblastogenesis. The positive effects on preosteoblast eventually result in increased bone nodule formation, a hallmark of* in vitro* osteogenesis [80, 85, 88].

Age-related bone loss is generally associated with osteoblast insufficiency relative to the adipogenesis, which is responsible for the progressive adiposity often observed in osteoporosis. The number of mature osteoblasts and adipocytes in bone marrow is influenced by the differentiation of the common mesenchymal progenitor cell towards one phenotype and away from the other. In contrast to the promoting role in osteoblast, strontium exhibits inhibitory role in adipogenesis of MSC [89]. In murine MSC cultures, strontium increased Runx2 expression and matrix mineralization and decreased peroxisome proliferator-activated receptor gamma 2 (PPARc2) expression and adipogenesis. This effect was associated with enhanced expression of the Wnt noncanonical representative Wnt5a and adipogenic modulator Maf and was abrogated by Wnt- and nuclear factor of activated T-cells (NFATc) antagonists, indicating a critical role for the Wnt and NFATc/Maf signaling in the switch in adipogenesis to osteoblastogenesis induced by strontium [89].

## 6. Molecular Basis of the Role of Strontium in Bone Forming Cells

### 6.1. Calcium Sensing Receptor (CaSR)

The CaSR belongs to subfamily 3 of G-protein coupled receptor family (GPCR) [90, 91] and can activate G*α*i and G*α*q/11 G-proteins, consequently resulting in decreased intracellular cyclic adenosine monophosphate (cAMP) level, the stimulation of phospholipase C*β*,inositol1,4,5-triphosphate, and the release of intracellular Ca^2+^ [90]. The CaSR is physiologically expressed at high level in the parathyroid chief cells and senses changes in extracellular Ca^2+^ level, leading to an adjustment in the release of the PTH [90]. Similarly, CaSR can sense other divalent and trivalent cations, including Sr^2+^ because of its similar atomic and ionic properties to Ca^2+^.

Experimental evidences show that strontium acts on osteoblasts through the CaSR, leading to the activation of MAPK signaling and consequently cell replication. In addition, strontium increases OPG and decreases RANKL expression in osteoblastic cells via the CaSR [92]. However, CaSR does not appear to be the only receptor involved in the effects of strontium on osteoblasts as increased cell replication and decreased apoptosis were still observed in osteoblasts from mice deficient for CaSR when exposed to strontium, indicating that other cation-sensing receptors are functioning to sense extracellular Sr [93]. Among these receptors are GPRC6A, a GPCR that is closely related to the CaSR and senses extracellular divalent cations [94]. The functional involvement of this and other cation-sensing receptors in the response to strontium remains to be further determined.

### 6.2. Fibroblast Growth Factor Receptor (FGFR)

As mentioned above, strontium also stimulates osteoblast cell growth through the CaSR-independent molecular mechanism. In this regard, a selective inhibitor of FGFR was able to slow down cell growth induced by strontium ranelate in osteoblastic cells, suggesting that the activation of FGFR is a new potential mechanism by which strontium can stimulate osteoblastic cell growth. Activation of FGFR-dependent cell growth is also observed in response to other cations, suggesting that activation of FGFRs may be a new cation-sensing mechanism in osteoblasts [95].

### 6.3. Ras/MAPK Pathway and Ras/Akt Pathway

Our own recent work demonstrated that rat sarcoma viral oncogene homolog (RAS), an upstream regulator of MAPK and Akt, was activated by strontium treatment and siRNA-mediated Ras knockdown inhibited strontium-stimulated expression of osteogenic markers [96]. Mitogen-activated protein kinase (MAPK) can directly enhance osteogenesis through the phosphorylation and subsequent activation of Runx2 [97]. These studies suggest that strontium can promote osteogenic differentiation of MSCs through activating the Ras/MAPK signaling pathway and the downstream transcription factor Runx2 [96]. Akt kinase plays certain roles in cellular processes, including glucose metabolism and cell proliferation and apoptosis. Studies on mice have demonstrated that strontium increased the proliferation and reduced the apoptosis of osteoblast with activation of the Akt kinase-related pathway [98].

### 6.4. Prostaglandins (PGE2)

It has been confirmed that the effect of strontium on the proliferation and reduced apoptosis of osteoblasts could be neutralized by the selective inhibition of cyclooxygenase-2 (COX-2). The results also indicated that the positive effects of Sr ranelate on osteoblasts depend on the PGE2 production. Strontium induces murine MSCs to express COX-2 that leads to the increased PGE2 production, thus contributing to the increased differentiation of MSC into osteoblasts [99]. This may therefore be a new pathway that is activated by strontium in bone cells.

### 6.5. The Role of the OPG/RANKL System

The primary mechanism by which strontium reduces osteoclast number is to regulate the production of osteoprotegerin (OPG) by osteoblasts and the receptor activator of nuclear factor *κ*-B ligand (RANKL) [100], the two molecules that play essential roles in osteoclast differentiation. Osteoblast progenitors and osteoblasts express RANKL, a molecule that binds to the receptor activator of nuclear factor *κ*-B (RANK) on osteoclast precursors and thereby activates intracellular signaling, resulting in osteoclast differentiation. Osteoblasts can produce OPG, which functions as a decoy receptor for the RANKL and can thereby reduce the production of osteoclasts by inhibiting the differentiation of osteoclast precursors. A positive effect of Sr ranelate was observed on the OPG/RANKL ratio, causing an increased secretion of OPG and a simultaneous reduction of RANKL expression, leading to the suppression of osteoclastogenesis [101]. The increase in the number of OPG-producing cells and the decrease in RANKL expression thus constitute the important mechanism by which strontium exhibits a dissociating effect on the coupling between bone formation and resorption.

### 6.6. The NFATc Pathway

The involvement of the calcineurin (Cn)/nuclear factor of activated Tc (NFATc) pathway in bone development and bone remodeling was found in patients who were taking Cn inhibitors such as cyclosporine (CsA) and FK506, developed osteopenia, and had a higher incidence of fractures [102]. It is known now that NFATc1 plays an important role in both osteoblasts and osteoclasts [103]. NFAT includes five transcription factors, NFATc1 to NFATc4 and NFAT5, which are all involved in the differentiation of various cell types. Highly phosphorylated NFATc transcription factors are normally localized in the cytoplasm. Increased intracellular calcium levels activate calcineurin (Cn) and dephosphorylate NFATc1, leading to its nuclear translocation to regulate target genes [104]. NFATc1 is expressed in the process of osteoblast differentiation and bone formation [105] and has been shown to be an important signaling pathway involved in the role of strontium. Increasing strontium concentrations in the osteoblast environment activate Cn and subsequently the NFATc/Wnt signaling pathways in osteoblasts, leading to the increased replication and suppressed adipogenesis [89].

### 6.7. The Role of Wnt Signaling

It is reported that strontium promoted the translocation of *β*-catenin into the nucleus through the activation of the CaSR and subsequent activation of Akt-signaling in human osteoblasts [98]. In addition, strontium decreased the expression of sclerostin, an inhibitor of the canonical Wnt signaling that acts as a negative regulator of bone formation [98], providing another mechanism by which strontium modulates the Wnt signaling. Further evidences on the involvement of strontium in the Wnt pathway include that the treatment of murine osteoblasts with strontium increased the expression of Wnt3a and Wnt5a and *β*-catenin transcriptional activity. Inhibitors of the Wnt signaling, DKK1, and sFRP1, a soluble protein with homology to the Wnt-binding site of Frizzled proteins, decreased the Sr-induced expression of osteoblastic genes such as Runx2, ALP, and type 1 collagen [93]. The inhibition of the Wnt5a receptor Ryk and the signaling transducer RhoA were able to partially abrogate Sr-induced cell proliferation and osteoblastic gene expression [93]. Another study showed that Wnt5a was expressed at higher levels in the bone marrow of Sr-treated senescent SAMP6 mice compared to vehicle-treated mice and that it played a role in the differentiation of MSC upon Sr-treatment [89]. Therefore, strontium definitely produces positive effects in bone via the Wnt signaling.

### 6.8. Insulin-Like Growth Factor

A recent study showed that an increase of the insulin-like growth factor (IGF-1) concentration was observed after six-month administration of Sr ranelate at a daily dose of 2.0 g [106]. This result suggests that IGF deficit plays a definite role in the development of postmenopausal osteoporosis and strontium administration may exert an advantageous influence on BMD increase.

In summary, multiple mechanisms are involved in strontium effects on bone forming cells. Once exposed to strontium, multiple intracellular signaling pathways and key molecules are activated and orchestrated to promote their survival, proliferation, and differentiation of osteoblasts, which in the meantime produce a series of inflammatory and osteoclast-regulatory factors that may eventually favor bone formation via coupling of both bone resorption and bone formation.

## 7. Strontium-Integrated or Substituted Biomaterials for Bone Regeneration

Tissue engineering is extensively applied in both research and clinical practice of skeletal disease and trauma. For tissue engineering, bioactive materials, with the properties of stimulating osteoblasts and calcimimetics in physiochemical behaviors, are preferential as an implant to facilitate healing or to compensate for bone defects, particularly for osteoporotic fractures where the conventional metallic implant is not applicable because of bone fragility and extremely low bone density. The key of a bioactive material is to form a continuous and highly reactive interface with the surrounding bone tissue to induce abundant bone formation and the ability to be structurally and mechanically compatible with bone tissue and eventually to be replaced by new bones. In some circumstances, it is also demanded that growth factors, drug, and ions are incorporated into such a material, which can in turn be slowly released as the material is biodegraded* in vivo*. As the role in stimulating osteoblast proliferation and differentiation becomes well recognized, strontium is widely applied in many bone regeneration biomaterials in various forms, which are briefly presented as below.

### 7.1. Strontium Integration into Bone-Supporting or Regenerating Biomaterials

In a study by Zhang et al. [107], strontium-containing mesoporous bioactive glass (Sr-MBG) scaffolds with controlled architecture and enhanced mechanical strength were fabricated using a three-dimensional (3-D) printing technique. The Sr-MBG scaffolds could combine the advantages of Sr-MBG such as good bone forming bioactivity, controlled ion release, and enhanced mechanical strength and thus has potential application in bone regeneration. polycaprolactone (PCL) is a resorbable polymer extensively used in bone tissue engineering owing to good structural properties and processability. Several studies reported that strontium-substituted bioactive glass (SrBG) was incorporated into PCL and fabricated into 3D bioactive composite scaffolds that exhibited better ability to promote osteogenesis than the bioactive glass without strontium [108, 109]. Strontium- (Sr-) substituted hydroxyapatite (HAP) scaffolds in the forms of nanopowders or microspheres were also reported to possess osteoconductive and osteoinductive properties and have the potential to repair bone defects caused by osteoporotic fractures [110].

The most important property of bone cement as inorganic filler in load bearing orthopaedic implants is good integration with host bone with reduced bone resorption and increased bone regeneration at the implant interface. Similar to integration into bioglass, strontium has been introduced into bone cement and the mechanical properties, crystallinic properties, and Sr ion release activities have been well evaluated. Sr-containing bone cement has been shown to promote early bone formation by prolonging the release duration of strontium while remaining the strength [111].

Biomimetic apatites could also be cosubstituted with Sr as well as other elements. In a study by Iafisco et al. [112], hydroxyapatites were cosubstituted with foreign ions such as Mg^2+^, CO_3_^2−^, and Sr^2+^ for starting materials for the development of nanostructured biodevices for regeneration of osteoporotic bone. Biological-like amounts of Mg and CO_3_ ions were inserted in the apatite structure to mimic the composition of bone apatite by the addition of Sr ions as antiosteoporotic agent. Based on the hypothesis that the combination of Si and Sr may have synergetic effects on osteoporotic bone regeneration, the porous Sr-substituted calcium silicate (SrCS) ceramic scaffolds combining the functions of Sr and Si elements were developed with the goals to promote osteoporotic bone defect repair [113]. Boron is known to play important roles in bone growth and maintenance, immune function, and psychomotor skills. Pan et al. reported that the incorporation of strontium significantly decreased the cytotoxicity that arises with the rapid dissolution of borate glass [114]. In addition, with the degradation of glass, it will not only render boron as a nutritional element, but also deliver strontium for new bone formation.

### 7.2. Strontium Substitution in Implant Coating Materials

Implants undergoing early instability or even subsidence correlate with an increased risk of aseptic loosening, subsequently requiring revision. A load-bearing orthopaedic implant therefore needs to possess the properties of well integration with host bone tissue and early fixation by osseointegration of the implant is indeed demanded during surgical practices. This is an even greater challenge during revision replacement surgery using the allograft implant because resorption of the allograft may exceed new bone formation and result in the instability of the prosthesis. Implantation of metal-based joint replacements often results in corrosion and particle release, initiating chronic inflammation leading onto osteoporosis of host bone. A compensative solution is the coating of metal implants with hydroxyapatite (HA) or the use of bulk bioglass. For this purpose, strontium could be incorporated into the material surface or applied in doping surface of an implant. Sabareeswaran et al. therefore tested the* in vivo* biocompatibility and bone healing of the strontium- (Sr-) stabilized bulk glass ceramics for short term implantation of up to 12 weeks in rabbit model and observed excellent healing, which is comparable to that seen during the use of a commercially available implant of HA-based bioglass alone [115]. A strontium-substituted nanohydroxyapatite (Sr-HA) coating, deposited onto porous implant surfaces, has the potential to enhance implant osseointegration [116]. In a cementless, experimental gap model in canine, Vestemark et al. compared a 5% strontium-doped HA bone graft extender with a nondoped HA extender and demonstrated that the extender with strontium doping could protect the allograft from fast resorption and increase gap healing, leading to the improved fixation of the implant, though the results of mechanical test were inconclusive, suggesting that strontium could contribute to reversing the imbalance of fast resorption of allograft and slower formation of new bone because of its anabolic and anticatabolic effects. In addition, it has been reported that a film of strontianite was formed on a bioactive surface of sodium titanate when exposed to a strontium acetate solution. This strontianite film enables the local release of strontium ions from implant surfaces and thus stimulates bone formation* in vivo* [117].

### 7.3. Strontium in Membrane Materials and Hydrogels

Membrane materials are particularly useful in guided bone regeneration. In this regard, the effects of a strontium hydroxyapatite- (SrHA-) containing membrane have exhibited higher elasticity and strength than the collagen membrane [110]. Meanwhile, slow strontium ion release was also confirmed to stimulate new bone formation.

Strontium has also been introduced into different hydrogels to be used in bone repair. A study reported that a bone tissue engineering approach in which arginine-glycine-aspartic acid- (RGD-)modified alginate hydrogels are crosslinked with bioactive strontium, zinc, and calcium [118]. It was further shown that strontium gels made with a high percentage of guluronic acid residues (high G) were degraded more slowly than those made with alginate rich in mannuronic acid (high M) and supported proliferation of osteoblast-like cells. After an initial burst, strontium release from alginate gels was steady and sustained, and the magnitude of release from high M gels was biologically relevant [118].

An amidated carboxymethylcellulose hydrogel enriched with Sr ions was evaluated for its effects of strontium released in the culture medium on osteodifferentiation. It has been shown that strontium released from the gel promotes the osteodifferentiation as shown by the increase of ALP activity, suggesting that the Sr-containing gel could represent a new strategy in bone tissue engineering.

## 8. Conclusion and Future Perspectives

Osteoporosis has become a serious and common public health problem. Aging is associated with impaired bone formation as a principal pathogenetic mechanism mediating bone fragility in osteoporosis. At the cellular level, physiology for individual osteoblast survival/growth, apoptosis, migration, and stress response are regulated through elaborated molecular feedback mechanisms. Although further analysis of such mechanisms is still mandatory in order to develop new therapeutics, it is certain that multiple pathways or molecules play important roles from different respects, some of which are emerging as therapeutic targets. Apart from currently proposed or trialed approaches to focusing on agonists/antagonists of certain osteoblastic signaling pathways, such as hPTH1-34 (teriparatide) and antisclerostin or anti-DKK1 antibodies, other strategies aiming at mobilizing skeletal stem cells and activating osteoblastic cell functions are anticipated to be a field worth of much exploring. Strontium, though currently thought as a relatively mild anabolic agent, is considered as a candidate in this respect due to its dual role in regulating osteoblastogenesis/osteoclastogenesis and osteogenesis/adipogenesis via its involvement in multiple pathways. Besides being able to be supplied systemically, the application of strontium is being much extended into various biomaterials and thereby tissue engineering strategies for the local bone lesions and defects.

Further, on the mounting evidences and rapid progress in revealing key molecular factors and signaling pathways regulating bone forming cells and osteoporotic conditions, a systems biology approach to coherently relating these factors with mathematical modeling would be particularly helpful to better understand and evaluate therapeutics. Future progress in this field will hopefully provide opportunities for exploring drug discovery.
